# Clinicians’ Concerns About Mobile Ecological Momentary Assessment Tools Designed for Emerging Psychiatric Problems: Prospective Acceptability Assessment of the MEmind App

**DOI:** 10.2196/10111

**Published:** 2019-04-25

**Authors:** Christophe Lemey, Mark Erik Larsen, Jordan Devylder, Philippe Courtet, Romain Billot, Philippe Lenca, Michel Walter, Enrique Baca-García, Sofian Berrouiguet

**Affiliations:** 1 EA 7479 SPURBO Université de Bretagne Occidentale Brest France; 2 URCI Mental Health Department Brest Medical University Hospital Brest France; 3 IMT Atlantique Lab-STICC F-29238 Brest Brest France; 4 Black Dog Institute University of New South Wales Sydney Australia; 5 Graduate School of Social Service Fordham University New York, NY United States; 6 Inserm U1061, La colombière Hospital University of Montpellier Montpellier France; 7 Department of Emergency Psychiatry and Acute Care CHU Montpellier University of Montpellier Montpellier France; 8 Fondamental Foundation Créteil France; 9 Carlos III Institute Of Health CIBERSAM (Centro de Investigation en Salud Mental) Madrid Spain; 10 Department of Psychiatry Universitad Catolica Del Maule Talca Chile; 11 Department of Psychiatry General Hospital of Villalba Madrid Spain; 12 Department of Psychiatry University Hospital Rey Juan Carlos Mostoles Spain; 13 Deparment of Psychiatry University Hospital Infanta Elena Valdemoro Spain; 14 Psychiatry Department Autonoma University Madrid Spain; 15 Department of Psychiatry IIS-Jimenez Diaz Fondation Madrid Spain

**Keywords:** acceptability, feasibility studies, mobile applications, ecological momentary assessment, decision support systems, clinical, internet, outpatients, young adult, prodromal symptoms, mental health

## Abstract

**Background:**

Many mental disorders are preceded by a prodromal phase consisting of various attenuated and unspecific symptoms and functional impairment. Electronic health records are generally used to capture these symptoms during medical consultation. Internet and mobile technologies provide the opportunity to monitor symptoms emerging in patients’ environments using ecological momentary assessment techniques to support preventive therapeutic decision making.

**Objective:**

The objective of this study was to assess the acceptability of a Web-based app designed to collect medical data during appointments and provide ecological momentary assessment features.

**Methods:**

We recruited clinicians at 4 community psychiatry departments in France to participate. They used the app to assess patients and to collect data after viewing a video of a young patient’s emerging psychiatric consultation. We then asked them to answer a short anonymous self-administered questionnaire that evaluated their experience, the acceptability of the app, and their habit of using new technologies.

**Results:**

Of 24 practitioners invited, 21 (88%) agreed to participate. Most of them were between 25 and 45 years old, and greater age was not associated with poorer acceptability. Most of the practitioners regularly used new technologies, and 95% (20/21) connected daily to the internet, with 70% (15/21) connecting 3 times a day or more. However, only 57% (12/21) reported feeling comfortable with computers. Of the clinicians, 86% (18/21) would recommend the tool to their colleagues and 67% (14/21) stated that they would be interested in daily use of the app. Most of the clinicians (16/21, 76%) found the interface easy to use and useful. However, several clinicians noted the lack of readability (8/21, 38%) and the need to improve ergonometric features (4/21, 19%), in particular to facilitate browsing through various subsections. Some participants (5/21, 24%) were concerned about the storage of medical data and most of them (11/21, 52%) seemed to be uncomfortable with this.

**Conclusions:**

We describe the first step of the development of a Web app combining an electronic health record and ecological momentary assessment features. This online tool offers the possibility to assess patients and to integrate medical data easily into face-to-face conditions. The acceptability of this app supports the feasibility of its broader implementation. This app could help to standardize assessment and to build up a strong database. Used in conjunction with robust data mining analytic techniques, such a database would allow exploration of risk factors, patterns of symptom evolution, and identification of distinct risk subgroups.

## Introduction

### Optimizing Data Collection

Over the last decade, the field of medicine has evolved toward greater digitization of data in order to improve coordination and continuity of care [1,2]. Information and communication technologies have brought computer science into medical units; medical data collection software is now abundant, and its use is expanding into a variety of settings [2,3]. However, many clinicians view this new reliance on technology as an added burden to their existing workload [2,4-6]. Despite some attempts to standardize practices, no single tool has been widely incorporated into routine use [4,7].

Data collection tools are often based on information stored as uncoded free text. The diversity of practices and theoretical orientations (interindividual variability) of each practitioner brings a high variability that could be a barrier to the processing of such data for clinical decision-making purposes and research purposes [4]. Intraindividual variability is affected by many factors, such as time, personal subjective factors [4], patient interaction, consultation environment, and symptoms presented by the patient [8], and this variability is greatly increased when there is no data collection frame [9]. Several studies have shown that using standardized semistructured interviews can improve the quality and completeness of data collection [9,10]. In addition, use of standardized semistructured interviews also facilitates the collection and use of high-quality data for research purposes without increasing the clinical workload, facilitating prospective observational studies and clinical studies [11].

Thus, it is important to optimize data collection as part of routine clinical practice and decision making, to standardize the collection of a common minimum dataset, and to develop electronic data collection platforms to support these activities [12].

### Objective

Our early mental disorder detection program aims to identify emerging psychiatric disorders in young outpatients. A standardized assessment is performed by a member of the clinical team to detect individual risk factors for a psychiatric disorder and to inform care. The objective of this study was to assess clinician acceptability of using a computer clinical data collection tool during a consultation. Secondary objectives of this study were to explore (1) the technical feasibility of using a connected tool during a consultation (accessibility, compatibility with the tools available in the university hospital), (2) the effectiveness of a computer interface for data collection (ease of use, ergonomics), and (3) the subjective experience of caregivers using the electronic health (eHealth) platform.

## Methods

### MEmind App Description

In partnership with the University of Madrid, we developed a Web app (MEmind) to allow the collection of clinical data in real time [13]. The tool allows clinicians to enter details into an electronic health record during a psychiatric evaluation, including sociodemographic details, clinical examinations, diagnoses, therapeutic factors, psychometric scales, and free-text observations.

The interface has been developed for cross-platform use on desktop computers, tablets, and smartphones, allowing use by health care professionals in different places in the practitioner’s workplace. Access is restricted by a password issued to the professional. The app can be customized to ambulatory practices and different mental health research protocols, and a wide range of relevant scales can be included in routine evaluations at the practitioner’s discretion.

For this study, we developed a semistructured interview script based on the standard evaluation form that the early detection team had previously used. The interview explores the patient’s sociodemographic background, the patient’s history, and the histories of their family members.

MEmind is also built to enable the patient to connect to another component, the personal health record, which they can access via a computer or smartphone to enter data in an ecological momentary assessment (EMA) view. Indeed, clinical assessment in psychiatry is usually based on findings from brief, regularly scheduled, in-person appointments. Although critically important, this approach reduces assessment to cross-sectional observations that miss essential information about disease course and are subject to recall bias. EMA involves repeated sampling of a person’s behaviors and experiences in real time, in their natural environment. Patient self-monitoring can rely on EMA procedures and lead to participatory medicine [11]. EMA has been successfully used for real-time self-reporting of symptoms and behavior in patients with anxiety disorders or suicide ideation. Given that psychiatry clinicians have previously relied exclusively on clinical interviews for diagnosis and treatment, the field could deeply benefit from this new source of data collected in real time covering information about the patient’s health state between visits. Mobile phones are generally kept on at all times and carried everywhere, making them an ideal platform for the broad implementation of EMA technology. EMA has a number of advantages. First, it gives the clinician insight into the contingencies of experience and mental states, based on prospective data. Second, EMA observations have, contrary to clinical interactions, ecological validity, reflecting real-life variation in response to real-life challenges [14]. These data can also be compared with the data collected during in-person visits and stored in regular eHealth reports or a Web-based data collection tool. We did not explore the use of EMA in this study, which focused on the clinicians’ experiences.

### Study Design

This study was a prospective acceptability study, designed to assess clinicians’ acceptability of the interface in routine clinical use. We asked clinicians from the 4 community psychiatry departments within Brest University Medical Hospital (France) to participate between June 31 and August 31, 2015. All clinicians working with adolescents and young adults were invited to participate by email or phone and gave their signed consent before taking part in this study. The only exclusion criteria were the clinician’s inability to use the computer tool and declining to participate.

After clinicians provided consent and were introduced to the study by an investigator, they were shown a video tutorial [15] to describe the functioning of the Web app. We then presented a video showing a first consultation with a patient presenting with emerging psychiatric concerns. This video reproduced the context of a consultation with a new patient, with their consent for the video to be used for this research purpose [16,17]. We asked clinicians to collect clinical data directly using the semistructured interview with the connected tool after or during the viewing. For this study, no patient clinical data were stored. The patient who appeared on the video agreed to having his image used for educational and research purposes.

**Figure 1:**
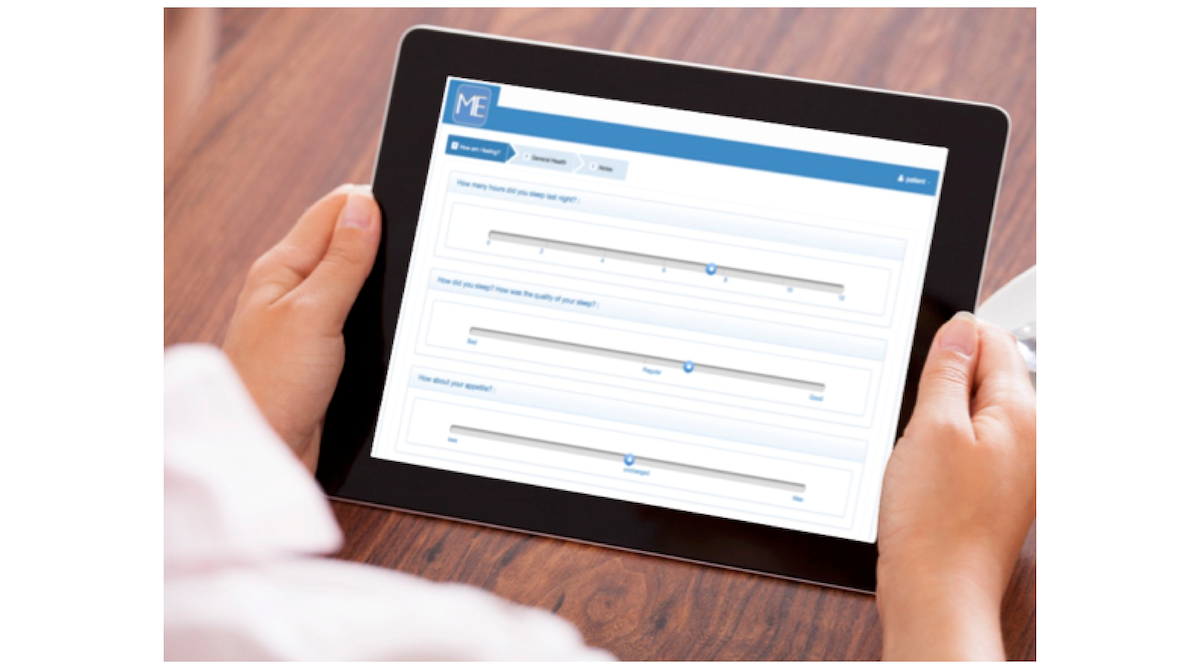
View of the connected tool.

We then evaluated the acceptability and technical feasibility of the app with an anonymized paper questionnaire (Figure 1).

We explored technical feasibility at each stage of the process. The questions focused on ergonomic, technical, and ethical issues, and the app’s possible use in routine practice. We measured acceptability using a 10-item paper self-questionnaire (Multimedia Appendix 1 shows the French version and Multimedia Appendix 2 shows an English translation) based on the Acceptability E-scale [18], which is a generic and validated questionnaire that can accurately evaluate satisfaction with a broad range of eHealth systems. This scale has been validated in French [19]. Participants completed the questionnaire after using the Web tool. The questions covered the technical feasibility and practical use of the tool, with each item graded using a 5-point Likert scale [20,21]. Additional questions centered on participants’ thoughts about daily use of computerized tools and their own habits. They were also asked to provide sociodemographic information about their age and sex. Depending on the question, other answers were given as either “yes” or “no,” as a choice between various options, or as free text [22].

We assessed the acceptability of the interface using a question from the Net Promoter Score, “Would you recommend this tool to your colleagues ?” This question is conventionally used in satisfaction studies and is a validated tool for this type of study [21].

Questionnaire response was anonymous. Participants submitted their data to a nonparticipating research nurse to preserve anonymity and reduce the risk of inadvertent breach of confidentiality. We digitized the answers of the self-questionnaire using double data entry to avoid transcription errors before statistical analysis. The results were described as percentage positive response.

### Statistical Analysis

We tested associations between categorical variables using a chi-square test of independence on the contingency tables and exact Fisher tests. *P* values are reported throughout the text when needed. We performed factorial analysis through a multiple correspondence analysis. This method is suitable for survey analysis, as it projects an initial set of qualitative variables in a factorial space where each dimension is a combination of the initial variables. We assessed the association between the initial variables (ie, the survey questions and the answer modalities) and the reduced dimensions with hypothesis testing.

The study was conducted according to French data processing requirements and the World Medical Association Declaration of Helsinki [23]. This study received a favorable ethical opinion from the Brest Medical University Hospital Ethics Committee.

## Results

### Participants’ Use of New Technologies

We invited 24 clinicians, and 21 (88%) agreed to participate. Of these, 12 (57%) were between 25 and 45 years old. Table 1 presents the characteristics of the participants’ use of new technologies.

Most practitioners regularly used new technologies, 76% (16/21) used a smartphone, and 95% (20/21) connected daily to the internet, 70% (15/21) at least 3 times a day. However, only 57% (12/21) felt that they were proficient with computers.

### Participants’ Views on the App

A total of 18 (86%) of the clinicians stated that they would recommend the Web app to their colleagues for data collection purposes. We found that 67% (14/21) felt that routine use in specialized assessments would be of interest, but when the subgroup analysis excluded practitioners who were only “a little” or “not at all” familiar with the computer tool, the acceptability of the software was 86% (18/21). Through a subgroup analysis, the acceptability of the tool thus increased significantly. We also performed a subgroup analysis according to the age of the participants (>55 years vs ≤55 years). An increase in age was not correlated with a decrease in acceptability of the interface (*P*=0.47).

Most clinicians (16/21, 76%) found the interface easy to use and useful. Table 2 shows data on the use of the tool. The enthusiasm for the computer tool didn’t depend on the age range of the clinicians. Although most participants had a positive experience with the platform, 2 of them (10%) found it not very useful, especially because 1 of them had encountered difficulties in connecting.

**Table 1:** Participants’ use of new technologies.

Characteristic		n (%)
**Frequency of internet use**		
	Less than once a week	0 (0)
	Between 5 and 10 times a week	1 (5)
	Once a day	5 (25)
	3 times a day	2 (10)
	More than 3 times a day	12 (60)
**Internet familiarity**		
	Not familiar at all	1 (5)
	A little familiar	2 (10)
	Moderately familiar	6 (30)
	Quite familiar	10 (50)
	Very familiar	1 (5)

**Table 2:** Participants’ views on use of the MEmind Web app.

Response	Question item category, n (%)					
	Ease of use	Usability	Understandability	Time to complete data entry	Completeness	Usefulness
Very satisfied	13 (62)	5 (24)	3 (14)	2 (10)	2 (10)	5 (24)
Somewhat satisfied	5 (24)	12 (57)	10 (48)	14 (67)	13 (61)	11 (52)
Very satisfied or somewhat satisfied	18 (86)	17 (81)	13 (62)	16 (76)	15 (71)	16 (76)
No opinion	2 (10)	1 (5)	3 (14)	1 (5)	1 (5)	2 (10)
Somewhat dissatisfied	1 (5)	3 (14)	5 (24)	2 (10)	5 (24)	2 (10)
Very dissatisfied	0 (0)	0 (0)	0 (0)	0 (0)	0 (0)	0 (0)

Several clinicians noted the lack of readability (8/21, 38%) and the need to improve the ergonomics (4/21, 19%) of the navigation interface within the various submenus. Several improvements were proposed to facilitate navigation and improve the ergonomics in the light of the free comments of some practitioners. Clinicians suggested several options: distributed tabs, drop-down menus, and a more compact presentation.

In general, practitioners appreciated the tool and considered that connection was easy (18/21, 86%), that usability was good (17/21, 81%), and that completing data entry was not time consuming (16/21, 76%). Chi-square tests confirmed the associations between participants’ level of satisfaction and the tested criteria (*P*=.01).

In the free observations that the clinicians could provide in the questionnaire, we noted that many of them showed an interest in this new tool. They pointed out that the app made it possible to standardize the collection of certain fundamental data by providing a framework that was sufficiently flexible. Others noted the lack of completeness and suggested enhancing the interface with additional scales and complementary articles. A major point of interest was that the interface provided secure access to patient data on different sites, reduced the redundancy of examinations, and made the best use of the very large amount of data collected during consultations.

### Participants’ Views on Online Patient Data Storage

Regarding the storage of patient information on the internet, Table 3 presents the distribution of the opinions of the psychiatric clinicians who participated in the study. This was the only question about which several clinicians did not wish to express an opinion. Together with those who selected the option “no opinion,” 52% (11/21) did not give any opinion on the storage of patients’ medical information on the internet. This is consistent with previous reports of physician concerns with online medical data storage [3].

**Figure 2:**
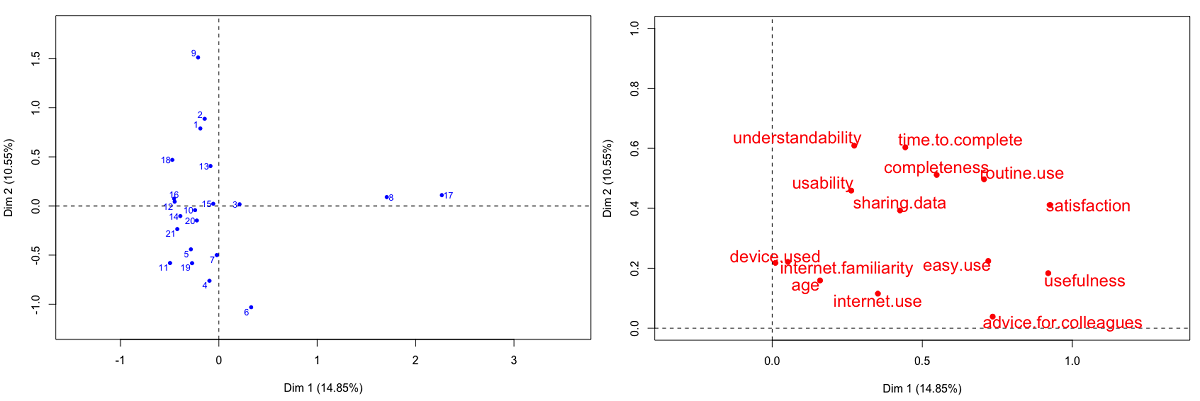
Multiple correspondence analysis. Projection of the variables onto the factorial map (left) and projection of the clinicians’ answers (right). Only the 2 first dimensions (Dim 1 and Dim 2) are represented.

A multiple correspondence analysis gave a synthetic view of the answers and the links between the satisfaction criteria. Figure 2 shows a 2-dimensional representation of the answers. The proximity between the points reflects the association between the survey answers. The results highlight some relevant trends: satisfaction was strongly linked to the tool completeness and a possible routine use, as well as ease of use and usefulness (dimension 1 on the x-axis). Understandability, usability, and time to complete data entry are also grouped as common criteria, expressing the ergonomics of the tool. Participants’ experience with the internet and use of devices to connect to the internet were also relevant factors for the acceptability of the tool.

**Table 3:** Practitioners’ views on online data storage, in response to the question “What do you think about storing patients’ medical data on the internet?”

Response	n (%)
Very dissatisfied	2 (10)
Somewhat dissatisfied	3 (14)
No opinion	7 (33)
Somewhat satisfied	4 (19)
Very satisfied	1 (5)
No answer	4 (19)

## Discussion

### Principal Findings

Our data support the feasibility of incorporating electronic health record data collection tools into routine clinical practice, to support the implementation of standardized data collection in outpatient services. The acceptability of a Web app for systematic clinical data collection was good among the clinicians surveyed. Specifically, more than 86% of clinicians would recommend the tool to their colleagues, 76% found it useful, and 67% would consider its routine use. Although only 57% of participants felt comfortable with computer use, 70% of them used it on a daily basis, and the acceptability of the Web app was very good. Importantly, this seems to reflect confidence in the tool and interest in these new ways of collecting information. There was a strong interest in this new type of interface, but there were also many issues related to medical confidentiality, and there was still some skepticism about online storage of patient data.

### Limitations

The participation rate in this study was 88%, and this sample was representative of the clinicians working in psychiatry at the Brest Medical University Hospital. However, the acceptability study we conducted had several limitations. The number of participants was small, and replication of the results on a larger scale in an everyday practice would be beneficial. Moreover, the semistructured interview used for this study followed the consultation model used for evaluations but did not contain psychometric scales or new items. Some participants who were more enthusiastic about the tool proposed adding new data to collect to the interface. It would be interesting to assess the acceptability of a tool that would change practices and the data collected, for example, by adding clinical scales or items that are not usually sought after. Indeed, clinicians are often reluctant to adopt tools that change the practices they have built over the years [3]. It seems, however, that the possibility of adding free text allowed clinicians to feel that they retained control over the tool, a concern that had already been identified in the literature [6].

The only elements that affected acceptability were technical aspects concerning ergonomics or difficulties of use related to connection problems at the time of testing. Age did not seem to have any influence on acceptability, whereas familiarity with computer tools did.

Several criticisms were stated regarding the readability of the interface. These anomalies did not prevent the use of the interface, which was simple but sometimes confused the user. Nevertheless, technical changes have to be made to correct display problems (tab alignments, page resizing. and title placement) and to make navigation within the interface more fluid, in particular by adding drop-down menus. These data are consistent with recent results showing the influence of ergonomic aspects on user experience and acceptability [17].

The study of the routine use of the interface would complete these results. It could also be interesting to test the use of such an interface by patients in their living environment. This may offer a new way of dealing with the symptoms they are reporting [13,24]. It is possible that future medical data tools may allow data to be collected during consultations and also on an ongoing basis in the patient’s living environment, using a combination of electronic medical records and EMA. This would allow clinicians to monitor the evolution of symptoms in their naturalistic setting [25,26]. The use of standardized questionnaires allows for homogeneous collection of clinical data for an informed medical decision. This also makes it possible to consider the use of health record databases for research or collaborations.

### Data Processing, Interest, and Ethical Issues

The growing use of tools dedicated to the collection of medical data, computerized medical health records, and communication tools is raising many ethical issues concerning confidentiality. This concern was reflected in the responses given by participants. However, this was the only question that more than one-third of clinicians did not answer, which exacerbates this concern.

Medical health data are being computerized within a cultural context of mistrust toward new technologies. The great ease of interpersonal communication and the flow of information entails a feeling of insecurity among individuals regarding confidentiality of these data [27]. Indeed, the use of computer tools makes it possible to heighten mistrust of transmission of information and the fear that personal data could be stolen [28]. The history of computer communication systems shows that, despite precautions taken by program developers, it is difficult to prevent data from being intercepted [29]. This is the most challenging question for clinicians: 33% of our participants did not express an opinion on the storage of data on the internet. If the participants who did not answer this question are included, the percentage increases to 42%. However, those who expressed an opinion were equally divided between satisfied (24%; very satisfied 5%, somewhat satisfied 19%) and dissatisfied (24%; somewhat dissatisfied 14%, very dissatisfied 10%). These results reflect a certain caution among the clinicians who did not seem to be opposed to the use of connected tools, but may have been concerned about engaging in a process they did not understand well.

There is thus a risk of revealing confidential information through cross-checking of data from various computer files and a risk of infringement of individual freedoms to the benefit of certain organizations, in particular administrative, financial, or insurance companies [29-31]. On the other hand, information technology tools are not always reliable in their handling (complexity of procedures, equipment breakdown or theft, loss or alteration of files, viruses, etc). Computer security can create anxiety for both the professional and the patient, particularly with respect to privacy issues. We found this concern among clinicians who took part in this acceptability study.

It is in the public interest to preserve citizens’ confidence in the confidentiality of the health care system. Particular attention must be paid to combining the use of information technology tools with the care of patients. In France, this concern is reflected in the legislative provisions of the law concerning the secrecy of health information and health data hosts [32-34]. These texts state that electronic medical records contain medical information and as such should be regarded as private and confidential. It also seems necessary to question the ownership of and access to the data collected. Do the data belong to the patient, the state, or the company that hosts the data or develops the interface? These questions also raise the possibility of using data collected in daily practice for research without explicit patient consent. The security of computerized personal health information systems is therefore an ethical imperative.

### Conclusion

We describe the first step of the development of a Web app combining an electronic health record and EMA features. This online tool offers the possibility of assessing patients and integrating medical data easily into face-to-face situations. The acceptability of this app supports the feasibility of its broader implementation. This app could help to standardize assessment and to build up a strong database. Used in conjunction with robust data mining analytic techniques, such a database would allow exploration of risk factors, patterns of symptom evolution, and identification of distinct risk subgroups.

## Appendix

Multimedia Appendix 1 Questionnaire (French).

Multimedia Appendix 2 Questionnaire (English).

## Abbreviations

eHealth: electronic health
EMA: ecological momentary assessment
