# Supplementary material for: Clinicians’ Concerns About Mobile Ecological Momentary Assessment Tools Designed for Emerging Psychiatric Problems: Prospective Acceptability Assessment of the MEmind App
Source: J Med Internet Res. 2019 Apr 25;21(4):e10111. doi: 10.2196/10111 (PMC6658238; doi:10.2196/10111)
Supplement: Multimedia Appendix 1 [file jmir_v21i4e10111_app1.pdf]

Evaluation de l'acceptabilité d'un dispositif de recueil systématique de données cliniques à travers une plateforme de e-santé destinée aux soignants

Date :
Numéro du sujet :

Pour chacune des questions, choisissez votre réponse parmi les cinq propositions suivantes :

1 : Pas du tout satisfaisant
2 : Plutôt pas satisfaisant
3 : Pas d'opinion
4 : Plutôt satisfaisant
5 : Très satisfaisant

Que pensez-vous de :	
La facilité de connexion au site MEmind ?...	☐
La facilité de navigation au sein du logiciel ?...	☐
La lisibilité du site ?...	☐
La facilité de remplissage des différents items ?...	☐
L'exhaustivité de l'outil ?...	☐
Pensez-vous que cet outil puisse être étoffé avec d'autres informations cliniques ? Si oui, lesquelles ?	
L'utilité de cet outil ?...	☐
Précisez	
L'utilisation de ce type d'outil pour homogénéiser les évaluations dans le cadre de ces consultations ?.........	☐
Précisez	
L'utilisation de cet outil en routine dans le cadre de la CEVUP ?...	☐
Stocker des informations concernant les patients sur internet ?...	☐
Conseilleriez-vous cet outil à l'un de vos collègues dans le cadre de ce type d'évaluations ?	OUI☐ NON☐
Dans quelle tranche d'âge vous situez-vous ?	25-35 ans ☐ 45-55 ans ☐ 35-45 ans ☐ 55-65 ans ☐ > 65 ans ☐
Quelle est votre familiarité avec l'outil informatique ?	Pas du tout ☐ Un peu ☐ Moyenne ☐ Correcte ☐ Très bonne ☐
Quelle est votre familiarité avec internet, vous connectez vous ?	Moins d'une fois par semaine ☐ Entre 5 et 10 fois par semaine ☐ Une fois par jour ☐ 3 fois par jour ☐ Plus de 3 fois par jour ☐
Vous vous connectez à l'aide de :	Ordinateur fixe ☐ Ordinateur portable ☐ Tablette ☐ Smartphone ☐ Autres objets connectés ☐
