# Supplementary material for: Clinicians’ Concerns About Mobile Ecological Momentary Assessment Tools Designed for Emerging Psychiatric Problems: Prospective Acceptability Assessment of the MEmind App
Source: J Med Internet Res. 2019 Apr 25;21(4):e10111. doi: 10.2196/10111 (PMC6658238; doi:10.2196/10111)
Supplement: Multimedia Appendix 2 [file jmir_v21i4e10111_app2.pdf]

Assessment of clinician acceptability of using a computer clinical data collection tool during a consultation

Date :
Subject number :

<u>For each question, choose your answer from the following five proposals :</u>

1: Very dissatisfied
2: Somewhat dissatisfied
3 : No opinion
4 : Somewhat satisfied
5 : Very satisfied

What do you think about	
How easy was the MEmind web application for you to use ?.......	
How much did you enjoy using the MEmind web application ?.......	
How understandable were the questions displayed in the MEmind web application ?.......	
Was the amount of time it took to complete the MEmind web application ?.......	
How would you rate the comprehensiveness of the MEmind web application ?.......	
Do you think this tool can be expanded with other clinical information? If so, which ones?	
How helpful to you was the MEmind web application ?.......	
Please specify	
How would you rate your overall satisfaction with this web application ?.......	
Please specify	
How would you rate your willing to use the MEmind application for routine practice ?.......	
Have you ever been sharing patient personal data on the internet ?.......	
Would you recommend the MEmind web application to your colleagues ?	YES☐ NO☐
How old are you ?	25-35 ☐ 45-55 ☐<br>35-45 ☐ 55-65 ☐ > 65 ☐
How familiar are you with the computer tool ?	Not familiar ☐ Unfamiliar ☐ Somewhat familiar ☐<br>Quite familiar ☐ Very familiar ☐
How familiar are you with the Internet, do you connect ?	Less than once a week □ Between 5 and 10 times a week □ Once a day □ 3 times a day □ More than 3 times a day □
You connect using :	Personal computer □ Laptop □ Tablet □<br>Smartphone □ Other connected objects □
